# Establishment of a Model of Microencapsulated SGC7901 Human Gastric Carcinoma Cells Cocultured with Tumor-Associated Macrophages

**DOI:** 10.1155/2018/3767482

**Published:** 2018-04-02

**Authors:** Jin-Ming Zhu, Xiu-Lian Quan, Shi-Chao Han, Xue-Jun Fan, He-Ming Li, Shan-Shan Liang, Xi Chen, Ruo-Yu Wang, Xue-Ning Ji

**Affiliations:** ^1^Department of Oncology, Zhongshan Hospital, Dalian University, Dalian, Liaoning, China; ^2^The Key Laboratory of Biomarker High Throughput Screening and Target Translation of Breast and Gastrointestinal Tumor, Liaoning Province, China; ^3^Department of Gynaecology and Obstetrics, The Second Affiliated Hospital of Dalian Medical University, Dalian, Liaoning, China; ^4^Department of Oncology, Zunyi Medical University, Zunyi, Guizhou, China

## Abstract

The important factors of poor survival of gastric cancer (GC) are relapse and metastasis. For further elucidation of the mechanism, a culture system mimicking the microenvironment of the tumor in humans was needed. We established a model of microencapsulated SGC7901 human GC cells and evaluated the effects of coculturing spheres with tumor-associated macrophages (TAMs). SGC7901 cells were encapsulated in alginate-polylysine-sodium alginate (APA) microcapsules using an electrostatic droplet generator. MTT assays showed that the numbers of microencapsulated cells were the highest after culturing for 14 days. Metabolic curves showed consumption of glucose and production of lactic acid by day 20. Immunocytochemistry confirmed that Proliferating Cell Nuclear Antigen (PCNA) and Vascular Endothelial Growth Factor (VEGF) were expressed in microencapsulated SGC7901 cells on days 7 and 14. The expression of PCNA was observed outside spheroids; however, VEGF was found in the entire spheroids. PCNA and VEGF were increased after being cocultured with TAMs. Matrix metalloproteinase-2 (MMP-2) and matrix metalloproteinase-9 (MMP-9) expressions were detected in the supernatant of microencapsulated cells cocultured with TAMs but not in microencapsulated cells. Our study confirms the successful establishment of the microencapsulated GC cells. TAMs can promote PCNA, VEGF, MMP-2, and MMP-9 expressions of the GC cells.

## 1. Introduction

Gastric cancer is one of the most common malignancies and the second leading cause of cancer-related death worldwide [1]. Although many therapies are currently available for GC, the 5-year overall survival rate is only about 50% owing to tumor relapse and metastasis. Recent evidence suggests that the tumor microenvironment (TME) is critical for tumor progression and metastasis [2]. Tumor-associated macrophages (TAMs) are derived from circulating monocytes, which are the most abundant immune cells in the tumor microenvironment [3] and are subjected to an intense cross talk with tumor cells. Macrophages can be polarized by cytokines, chemokines, and growth factors which are produced by stromal and tumor cells [4]. Meanwhile, TAMs secrete lots of factors that induce the formation of a network in which tumor cells can benefit by receiving nutrients and migrating to other sites [5]. Thus, TAMs can facilitate cancer promotion, angiogenesis induction, and tumor cell migration and metastasis [6].

However, studies that performed* in vitro* culturing of tumor cells or TAMs have important limitations. Most tumor cells cultured* in vitro* are grown as monotypic cultures in two-dimensional (2D) conditions, which cannot simulate* in vivo* TME conditions [7]. In comparison, three-dimensional (3D) cell culture conditions enable tumor cells to establish cell-cell and cell-extracellular interactions, which are important elements in tumor signaling and modulating tumor responses to therapeutic agents [8, 9].

Microcapsules are spherical, with diameters in the range of 200–1500 *μ*m, and feature a biocompatible semipermeable membrane that permits the bidirectional diffusion of nutrients, secreted therapeutic products, oxygen, and waste but prevents the passage of high-molecular-weight substances into the microcapsule. So, microcapsules can be used as an immune-isolation device [10]. Microcapsules can provide contained environments in which tumor cells grow in a three-dimensional manner and become adjusted to the intravital environment of the host so that they can perform a cross talk with the extracellular matrix. The reaction between the extracellular matrix and alginate microcapsules enhanced cell proliferation by triggering a cascade of intracellular signaling events through cell-matrix interactions [11, 12].

The model of microencapsulated tumor cells is a short-time, simple, and relatively inexpensive assay. Currently, many studies on microencapsulated tumor cell models on breast cancer, pancreatic cancer, lymphoma, melanoma, and osteosarcoma cells have already been performed. However, the microencapsulated human GC cell model is still lacking.

Here, we established a microencapsulated human GC cell model, called the microencapsulated SGC7901 model, using alginate-polylysine-sodium alginate (APA). This model can be widely used for preclinical studies and it provides reliable evidence for clinical therapy in GC patients. We evaluated the biological characteristics of this model and the effects on the encapsulated cells upon cocultivation with TAMs.

## 2. Materials and Methods

### 2.1. Materials

The human GC SGC7901 cell line was purchased from the Cell Bank of Shanghai Institute of Cell Biology, Chinese Academy of Sciences. Encapsulation device: a high-frequency pulse microdroplet generator was provided by Dalian Institute of Chemical Physics, Chinese Academy of Sciences.

### 2.2. Cell Culture

SGC7901 cells were cultured in a T flask (75 cm^2^) in RPMI1640 medium (Gibco, Grand Island, NY, USA) supplemented with 1% (v/v) penicillin-streptomycin and 10% fetal bovine serum (FBS, Gibco) in an incubator at 37°C and 5% CO_2_ at 100% relative humidity. The growth medium was replenished as necessary.

### 2.3. Differentiation of Macrophages from Peripheral Blood Mononuclear Cells

Blood samples (15 mL) were collected from healthy donors. Peripheral blood mononuclear cells (PBMCs) were isolated according to a previous publication [13]. PBMCs were plated in three 6-well plates (1 × 10^6^ cells/well) and incubated for 3 h in RPMI1640 (Gibco) supplemented with 1% (v/v) penicillin-streptomycin and 10% FBS in an incubator at 37°C and 5% CO_2_ at 100% relative humidity. On the next day, Recombinant Rat Granulocyte Macrophage-Colony Stimulating Factor (rGM-CSF) (Rocky Hill, NJ) was added (1000 u/ml) in 2 mL of RPMI 1640 (Gibco) containing 10% FBS (Gibco). The cells were cultured for 7 days, and the medium was changed every 3 days. Macrophages were harvested on day 7.

### 2.4. Identification of Macrophages

The cell suspension was prepared when the macrophage was cultured for 7 days. The cells were plated in a 24-well plate and cultured for 72 h. Cells were then fixed with 4% paraformaldehyde for 30 min, incubated with 0.2% Triton X-100 (Gibco) for 20 min, and then blocked with 5% BSA for 30 min. Cells were washed three times with PBS for 5 min between the different steps above. The fixed cells were incubated with primary antibody against CD68 (ZSGB-BIO, Beijing, China) (1 : 100) at 4°C overnight and then incubated for 30 min with secondary antibody (Santa Cruz, Dallas, TX, USA) at room temperature in the dark. Cells incubated with PBS as a primary antibody served as the negative control. Stained cells were sealed with glycerol and observed with a confocal microscope (Olympus Optical Co., Ltd., Japan).

### 2.5. Cell Microencapsulation

The process for encapsulation of human tumor cells was based on the encapsulation technology provided by Dalian Institute of Chemical Physics. The encapsulation preparation was performed according to a previous publication [14]. SGC7901 cells were suspended at a final concentration of 4 × 10^9^ cells/L in sodium alginate. The suspension was then passed through a jet head droplet-forming device yielding spherical microdroplets (diameter: 600 *μ*m), with about 600–800 cells in each droplet. These microdroplets were formed into discrete water-insoluble gel spheres by contact with a lightly stirred solution of CaCl_2_. The next step was the formation of a semipermeable membrane on the surface of the gel beads induced by addition of polylysine (Sigma Chemical Co., St. Louis, MO). This was called polylysine-sodium alginate (PA) microencapsulation. By suspending the PA microencapsulation in a 1.2% low-viscosity sterile pharmaceutical-grade alginate, the APA was obtained. This process yielded microencapsulated cells containing viable tumor cells in liquid suspension. The microencapsulated cells were cultured in low sugar Dulbecco's modified Eagle's medium (DMEM; Gibco, Grand Island, NY, USA) containing 10% FBS and 1.25 mL/l gentamycin (Gibco). Images of the cells were obtained with an inverted phase contrast microscope (Olympus Optical Co., Ltd., Japan).

### 2.6. Coculturing the Microencapsulated Cells and Macrophages

The microencapsulated SGC7901 cells were cultured for 14 days and the macrophages were cultured for 10 days individually. By that time, the macrophages were resuspended at a cell density of 3 × 10^4^ cells/L. At last, the microencapsulated SGC7901 cells and the macrophages were cocultured together in a 6-well plate for 72 h.

### 2.7. MTT Assays

We plated microencapsulated HGC cells onto a 96-well plate (50 *μ*l cells and 200 *μ*l medium per well) and cultured cells for 14 days. We added 50 *μ*l of MTT (Jun Chuang Chemical Co., Shanghai, China) solution (filtered 5 g/L MTT solution in serum-free medium) per well, covered the cells with aluminum foil, and incubated the samples at 37°C and 5% CO_2_ overnight. The MTT solution was removed and 150 *μ*l of DMSO (Baofeng Chemical Co., Tianjin, China) was added to each well, followed by gentle shaking to dissolve the purple crystals in the well for 10 min at room temperature. The absorbency at 540 nm and 630 nm was measured in a lab-system microplate reader (Labsystems Co., Ltd., Finland). Three wells were examined every 2 days to obtain an average reading.

### 2.8. Glucose and Lactic Acid Readings

We transferred 150 *μ*l supernatant from three wells every 2 days from the 96-well plate, which contained 50 *μ*l microencapsulated HGC cells and 200 *μ*l medium per well. Then, we froze the samples at −20°C. The glucose and lactic acid in the supernatant were evaluated by a biosensor (Shandong Academy of Sciences).

### 2.9. BrdU Proliferation Assays

The microencapsulated cells were cultured on a slide in a 35 mm dish. Cells were labeled with 5-bromo-2-deoxyuridine (BrdU) (ZSGB-BIO, Beijing, China) (0.3 g/L) and incubated at 37°C for 30 min. The labeling medium was removed and cells were washed with 1x PBS three times, fixed with 70% alcohol, and then air-dried. Next, the following reagents were added in turn: (a) 1 mol/L HCl, cultured at 37°C for 30 min; (b) 0.1 mol/L sodium borate, pH 8.5, at room temperature for 30 min; (c) 0.2% Triton X-100, pH 7.4, at room temperature for 10 min; (d) 5% normal goat serum (ZSGB-BIO, Beijing, China) block for 30 min. Cells were incubated with primary antibody (ZSGB-BIO, Beijing, China) for BrdU (1 : 50) at 4°C overnight. The slides were washed with PBS three times and stained with biotin-labeled secondary antibody (ZSGB-BIO, Beijing, China) for 30 min, followed by processing using the SP immune-histochemical kit (ZSGB-BIO, Beijing, China) staining reagent for 30 min at room temperature. The slides were washed with PBS three times and mounted in SlowFade Antifade (Jun Chuang Chemical Co., Shanghai, China). Positive staining was determined as brown or yellow staining in the nucleus.

### 2.10. Immunohistochemical Staining

The cocultured microencapsulated cells were fixed in 4% paraformaldehyde and then were embedded in paraffin. Paraffin-embedded sections were cut into standard 5 *μ*m sections, deparaffinized in xylene, and rehydrated through graded alcohol solutions. Endogenous peroxidase was inactivated by immersing the sections in 0.3% hydrogen peroxide for 30 min at room temperature. The samples were washed three times with PBS-Tween (5 min each) and then incubated in 0.01% mol/L pH 6.0 sodium citrate buffer for antigen retrieval (microwave, 400 W, 15 min). Samples were cooled to room temperature and then sections were incubated with primary antibodies against PCNA (ZSGB-BIO, Beijing, China) and VEGF (ZSGB-BIO, Beijing, China) overnight at 4°C. PBS was used as the primary antibody for control. The sections were stained with secondary antibody and the SP immune-histochemical kit (ZSGB-BIO) as described above. Finally, the sections were counterstained with hematoxylin, mounted, and observed with a microscope (Olympus Optical Co., Ltd., Japan). PCNA and VEGF were detected in monolayer cultured cells and microencapsulated cells using the same method.

IHC results were independently evaluated by two specialized pathologists. The expression of PCNA protein was mainly observed in the nuclei of tumor cells and VEGF protein was observed in the cytoplasm. In the groups of microencapsulated cells and cocultured microencapsulated cells, the PCNA and VEGF expression levels were estimated by the percentage of positive stained cells and the staining intensity (0, negative; 1, weak; 2, intermediate; 3, strong).

### 2.11. Gelatin Gel Zymography

The supernatant of the cocultured microencapsulated cells was centrifuged at 2000 rpm for 10 min; next, it was frozen in −80°C. The supernatant of microencapsulated cells was used as a control. The supernatant samples (20 *μ*l) and sample buffer were mixed together and then were vortexed and allowed to settle for 10 min before loading into wells in the gel. The voltage was first set to 70 V until the protein was stacked appropriately and then increased to 125 V for 90 min. The gels were then washed in 30 min washes using 1% Triton in distilled water to remove any remaining sodium dodecyl sulfate in the gels, followed by rinsing in distilled water to remove the remaining Triton. The gels were placed in developing buffer for 30 min and then incubated in a water bath at 37°C for 18 h. The gels were developed the next day using Coomassie Blue R-250 for 30 min and then destained using a destaining solution. The bands were scanned using a gel scanner. Intensities were analyzed by ImageJ 1.46 program (NIH) [15].

### 2.12. Statistical Analysis

The Mann–Whitney test was used to identify differences in nonparametric variables for two independent groups using GraphPad 7.0 software. (A value of *P* < 0.05 was considered statistically significant.)

## 3. Results

### 3.1. Phenotypic Characterization and Activity of the Microencapsulated SGC7901 Cells

**Figure 1:**
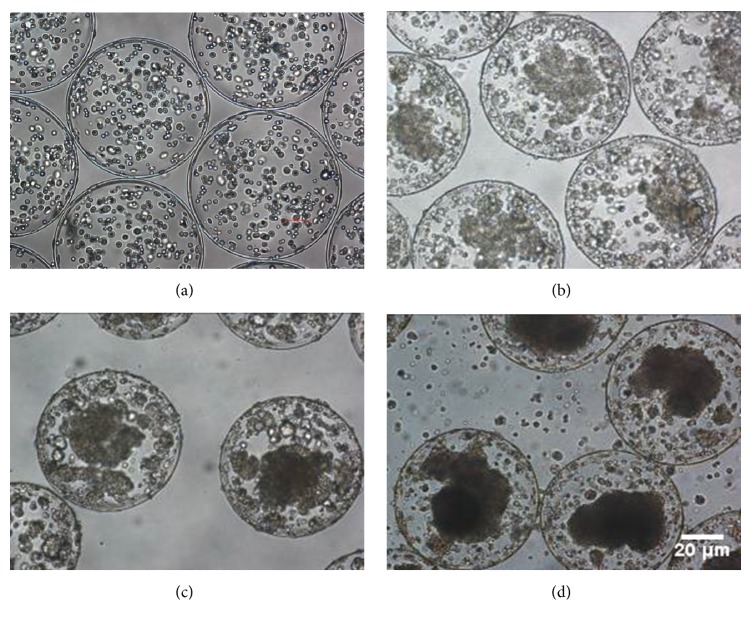
Phase contrast imaging of microencapsulated SGC9701 cells. (a) Microencapsulated cells on day 0 as control. (b) After culturing for 7 days, the cells tended to aggregate in spheres. (c) After culturing for 14 days, the spheres expanded rapidly and grew in three dimensions. (d) After culturing for 21 days, the sphere expansion slowed down and spheres showed necrosis in the center. Magnification: 200x.

Phase contrast imaging of the microencapsulated SGC7901 cells is shown in Figure 1. Microcapsules displayed a consistent appearance of a sphere with diameter of 500~600 *μ*m. The surface of the capsule wall was clearly smooth. The number of cells contained in each capsule was approximately 600–800. Microencapsulated SGC7901 cells were evenly distributed in the beads on the day of encapsulation and started aggregating into spheres upon culture for 48 h to 72 h (data not shown). Over the first 14 days of culture, we detected an increase in the diameter of spheres and cell concentrations (data not shown). The maximum diameter of the microencapsulated cells was 300 *μ*m.

**Figure 2:**
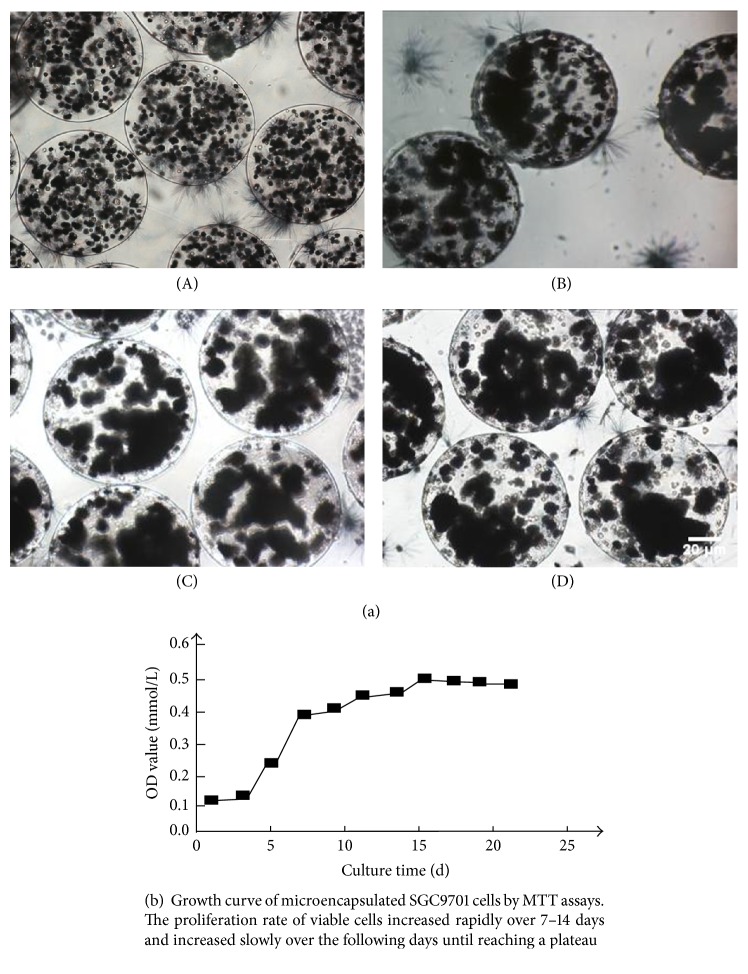


To evaluate the viability of cells in the spheres, we performed MTT assay (Figures 2(a) and 2(b)). MTT assay was an established method for monitoring cell viability based on mitochondrial activity. In our study, the MTT assay was proposed for the in situ quantification of the living cell density of microencapsulated SGC7901 cells. After 24 h of incubation with MTT, the bluish-violet crystals appeared on the outer layer of the cell spheres, indicating that the cells proliferated and aggregated to be a sphere in the early culturing time. We observed the maximum relative number of microencapsulated cells by day 14 of culture, and the cell number declined slightly over the following days (Figures 2(a) and 2(b)). These results show that the speed of cell death exceeded the rate of proliferation because of the limited nutrition and space in the late culturing time.

### 3.2. Metabolic Activity of the Microencapsulated Cells

**Figure 3:**
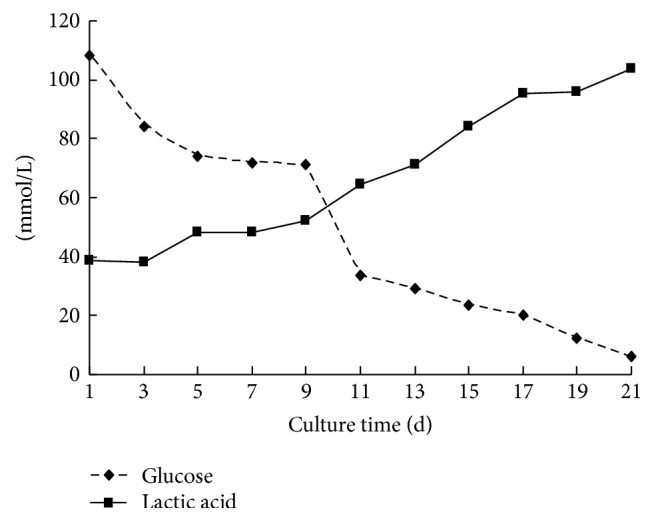
Concentration-time curve of glucose and lactic acid detected in the supernatant of microencapsulated SGC9701 cells.

To determine the metabolic activity of the cells in the spheres, we evaluated concentrations of glucose and lactic acid. We found that, over 21 days of culture of the cell spheres, these concentrations from the cultured microcapsules showed dynamic changes. By day 20, glucose levels decreased to 10 mmol/L compared with 110 mmol/L at day 1. In comparison, lactic acid increased from 40 mmol/L at day 1 to 100 mmol/L at day 20 (Figure 3). These data show that, with the increasing of the microencapsulated cells, the glucose was consumed while the acid was generated gradually. This suggested that the metabolic activity of the microencapsulated cells was the same as that of the tumor cells in the human body.

### 3.3. Proliferation of the Microencapsulated Cells

**Figure 4:**
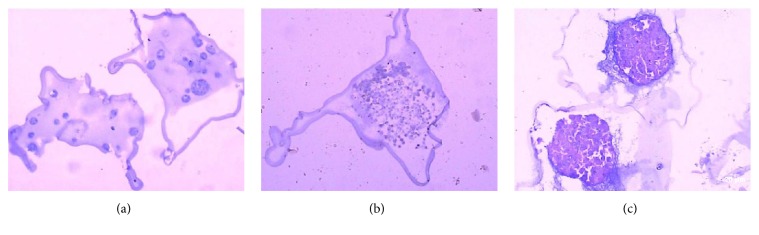
Proliferation of microencapsulated SGC9701 cells by BrdU staining. Brown or yellow nuclear staining indicates live cells. After culturing for (a) 7 days or (b) 14 days, the cells tended to aggregate into spheres. The entire microencapsulated spheres showed positive BrdU staining. (c) After 21 days, the staining color faded or the center of the cells showed no staining while the cells outside still showed staining.

To evaluate proliferation rates of the microencapsulated cells, we performed BrdU staining analyses (Figure 4). The microencapsulated cells in the spheres showed positive BrdU staining at both day 7 and day 14. By day 21, the level of BrdU staining in some spheres was very low or even no staining in the center, while the cells outside still showed strong positive BrdU staining. Together, this indicates that the microencapsulated cells in the beans have normal proliferation cultured in the first 14 days. The proliferation rate of the cells decreased in the following days because of the nonaffluent nutrition and space in the capsule. Thus, cell death was more than cell proliferation especially in the center of the spheres.

### 3.4. Identification of Macrophages

**Figure 5:**
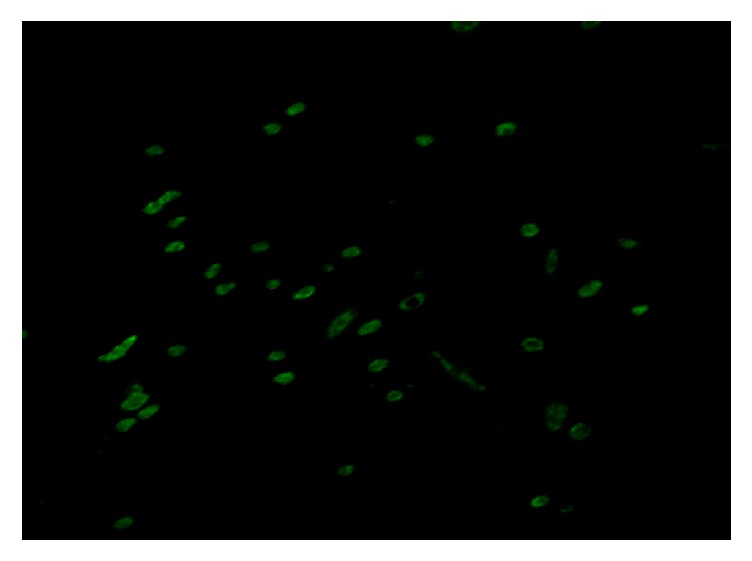
Identification of macrophages by CD68 immunofluorescence. Macrophages were differentiated from peripheral blood mononuclear cells and identified by CD68 immunofluorescence staining. Magnification: 100x.

CD68 was generally considered as a specific marker of macrophages, and therefore CD68 was used to identify TAMs in the macrophage culture system. Immunostaining for CD68 identified positive CD68-expressing cells as TAMs, which showed green fluorescence in the cytoplasm (Figure 5).

### 3.5. PCNA and VEGF Expression in Monolayer Cells, Microencapsulated Cells, and Microencapsulated Cells Cocultivated with Macrophages

**Figure 6:**
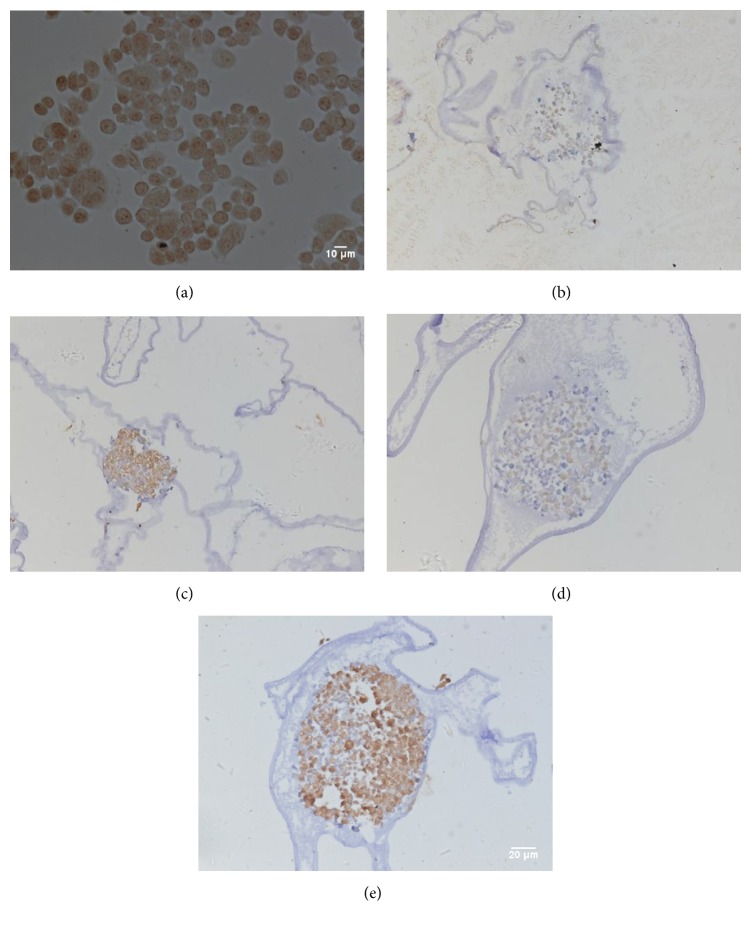
Expression of PCNA in the cells and spheres by H&E staining. Brown nuclei indicated positive PCNA staining. (a) Monolayer SGC9701 cells showed positive PCNA expression. (b, c) The microencapsulated cell spheres cultured for 7 days and 14 days: PCNA expression was observed throughout the entire spheres. (d) The microencapsulated cell spheres cultured for 21 days: PCNA expression was detected outside the spheres, but not in the center. (e) The microencapsulated cell spheres cocultured with macrophages for 3 days: the number and density of the spheres expressing PCNA were increased. Magnification: 200x.

**Figure 7:**
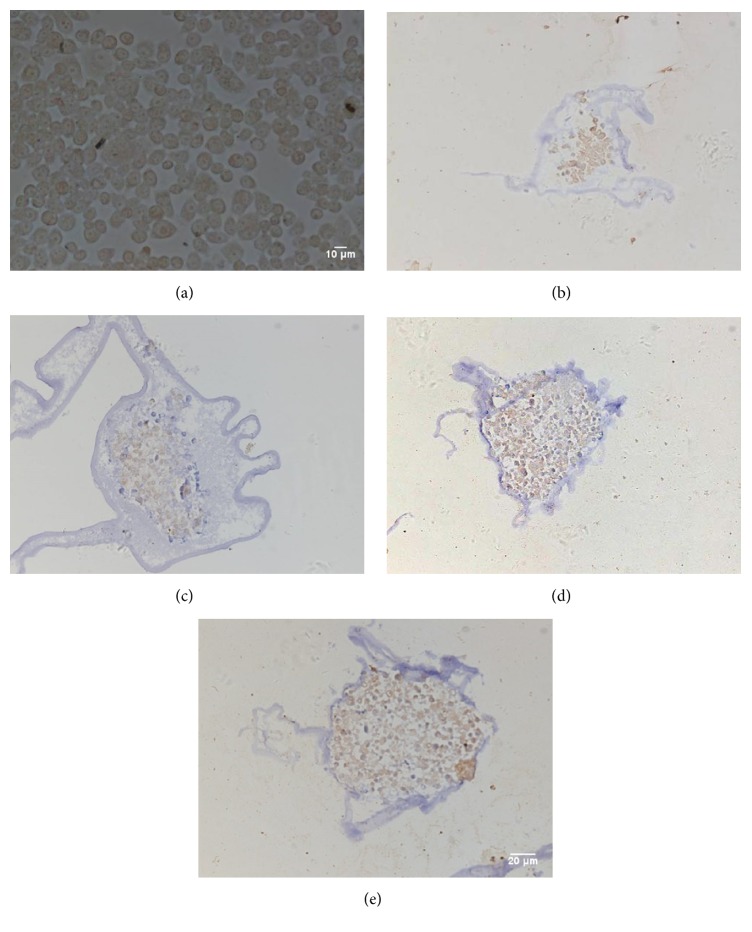
Expression of VEGF in the cells and spheres by H&E staining. Brown nuclei indicated positive VEGF staining. (a) Monolayer SGC9701 cells showed positive VEGF expression. (b, c, d) The microencapsulated cell spheres cultured for 7, 14, and 21 days: VEGF expression was observed throughout the entire spheres. (e) The microencapsulated cell spheres cocultured with macrophages for 3 days: the number and density of the spheres expressing VEGF were increased. Magnification: 200x.

As we all know, PCNA is considered as a specific marker for cell proliferation [16]. VEGF can induce the proliferation of vascular endothelial cells, promote the formation of new vessels, and increase vascular permeability. It contributes to metastasis and invasion of tumor cells. Previous clinical studies show that the higher the expression of VEGF in GC patients, the poorer the prognosis [17]. We next evaluated PCNA and VEGF expression in monolayer cells, microencapsulated cells, and microencapsulated cells cocultivated with macrophages. Both monolayer SGC9701 cells (Figures 6(a) and 7(a)) and microencapsulated SGC9701 cells showed expression of PCNA and VEGF. This result suggests that the microencapsulated SGC9701 cells have the same potential on proliferation and protein expression as monolayer cells. In microencapsulated SGC9701 cells that were cultured for 7 or 14 days, the cell spheres were not large enough and still increased rapidly. Thus, the entire spheres showed expression of PCNA and VEGF (Figures 6(b), 6(c), 7(b), and 7(c)). On day 21, the cell spheres were large enough, and due to the limited nutrients of the microcapsules, with the proliferation of the microencapsulated SGC9701 cells, the center gradually developed hypoxia and necrosis. PCNA was only found at the outside of the microencapsulated cell spheres, but not in the center (Figure 6(d)), which was a similar trend to BrdU shown above. However, VEGF expression was detected throughout the spheres (Figure 7(d)). The number and density of the microencapsulated SGC7901 cells expressing PCNA or VEGF were increased when SGC7901 cells were cocultured with macrophages (Figures 6(e) and 7(e)). The average values of PCNA and VEGF expression in microencapsulated groups were 52%, 40%, 78%, and 80% in the cocultured group (*P* < 0.05). Meanwhile, the semiquantitative expressions of PCNA and VEGF were significantly different between microencapsulated culture and coculture with macrophages based on staining intensity (*P* < 0.05). Together, these results show that the expression of PCNA and VEGF in the microencapsulated cells is consistent with that in the monolayer cells. TAMs can promote PCNA and VEGF expression of the microencapsulated SGC9701 cells.

### 3.6. MMP-2 and MMP-9 in Microencapsulated Cells Cocultured with Macrophages

When the macrophages were induced into the tumor microenvironment, MMPs would be produced. MMPs play important roles in the responses of cells to their microenvironment, by effecting proteolytic degradation or activation of cell surface and extracellular matrix (ECM) proteins, which facilitate tumor cells proliferation, differentiation, migration, and survival [18].

**Figure 8:**
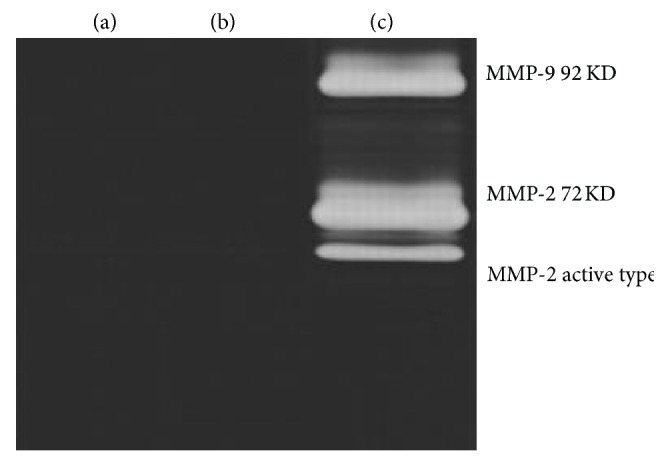
MMP-2 and MMP-9 expressions were detected by gelatin gel zymography. MMP-2 and MMP-9 expression in microencapsulated SGC7901 cells (a), macrophages (b), and microencapsulated SGC7901 cells cocultured with macrophages (c).

Therefore, we next evaluated the levels of MMP-2 and MMP-9 in cells (Figure 8). Expression of MMP-2 and MMP-9 was not found within the supernatant of microencapsulated SGC7901 cells or macrophages cultured alone. However, MMP-2 and MMP-9 were detected in the supernatant of microencapsulated SGC7901 cells cocultured with macrophages. These data indicate that TAMs can promote the expression of MMP-2 and MMP-9 in microencapsulated SGC7901 cells because of the cross talk in the TME.

## 4. Discussion

Metastasis and drug resistance are the leading causes of death in GC patients. Currently, basic experimental studies are mostly dependent on GC cell two-dimensional culture* in vitro* and xenotransplantation* in vivo*. Both of these methods have advantages and disadvantages in research. For example, tumor cells in animal xenograft models can grow in 3D conditions and also exist within a specific microenvironment that is similar to that observed in humans. However, animal experiments are relatively expensive, delicate, and highly susceptible to infection, and breeding and experimental conditions are strict. In contrast, although monolayer cells are more cost-effective, these cultured cells do not establish the special TME. Hence, there is an obvious gap between cell culture experiments and clinical application. Therefore, in our study, we aimed to establish a transitional culture model between the* in vivo* animal model and* in vitro* monolayer cell culture.

Here, we established a microencapsulated human GC cell model using the APA technique. The microencapsulated cells would be efficiently protected by the outer layer of the microcapsule and could undergo interaction with TMEs in the process of growth and activity. Moreover, prior studies have confirmed that microencapsulated tumor cells are more viable than monolayer cells, and the stability of tumor-associated genes is not affected [19]. In our study, the microencapsulated GC cells could grow in clusters within 48–72 h to form spheres. The maximal diameter of the spheres increased to 300 *μ*m when culturing for 14 days and stopped increasing in the following culturing days. The cells in the microencapsulated spheres also showed a strong proliferation activity, and the metabolic curves of glucose and lactic acid were consistent with cell proliferation. Furthermore, in our study, the expression of PCNA and VEGF was detected both in the monolayer cell culture and in the microencapsulated cell culture. PCNA and VEGF are related to cell proliferation, metastasis, and invasion of the tumor cells [16, 17]. According to a previously published paper, the microencapsulated MCF-7 cell model was stably expressed with PCNA and VEGF in a single cell 3D culture. Both of these proteins were demonstrated as potential specific markers for the evaluation of basic biological functions in both the monolayer cell culture and the 3D microenvironment [20]. Therefore, we propose that the basic biological function of the tumor cells was still preserved after establishment of the microcapsules and the microencapsulated SGC9701 cell model. In further studies, we will detect more proteins to prove this.

The tumor is not composed only of the cancer cells. Instead, it is composed of heterogeneous tumor cells, endothelial cells, immune cells, smooth muscle cells, fibroblasts, and macrophages [21, 22]. There is also a dynamic and mutualist relationship between tumor cells and the surrounding stroma [23]. Moreover, tumor cells can secrete several factors that modify the surrounding stroma. The extracellular matrix is responsible not only for the structural support of the cells but also for storing important signaling molecules, such as chemokines [24]. This forms a special TME that can determine the malignant phenotype of tumor cells and promote tumor metastasis [25, 26].

Macrophages are differentiated from mononuclear cells which are the principal members of inflammatory cells in tumor stroma. These cells can be stimulated into TAMs under a low oxygen environment or by the chemokines and growth factors secreted by tumor tissues. They have an extremely important action for tumor progression [27]. MMPs, a family of proteases secreted from myofibroblasts and tumor cells, are important for remodeling of the matrix and aid in the migration and invasion of tumor cells [26, 28].

In our study, the microencapsulated SGC7901 cells model can provide a TME between the tumor cells and the matrix due to the special microcapsule. When the microencapsulated SGC7901 cells were cocultivated with TAMs, we detected the expressions of MMP-2 and MMP-9 but not in microencapsulated SGC7901 cells cultured alone. In addition, an increased level of PCNA and VEGF was observed in the cocultured group compared with microencapsulated SGC7901 cells cultured alone. Thus, we presume that TAMs and microencapsulated cells that grow in three dimensions interact with each other to form a special microenvironment, which may be similar to the TME observed in the human body. We speculated that TAMs could facilitate the invasion and metastasis activity of GCs in the spheres by promoting the expression of PCNA and VEGF.

In conclusion, here we established a transitional* in vitro* 3D culture model, between* in vivo* animal models and* in vitro* single cell culture. The cells in this model showed normal growth and metabolism and stable protein expression. This microencapsulated SGC7901 cell model could form a TME which is similar to human while the monolayer cell culture could not. This model might provide a relative stable research platform for further study of the interaction between human gastric cancer cells and the host microenvironment.
